# Editorial: RNA and RNA modification in the pathogenesis, diagnosis and treatment of cancers

**DOI:** 10.3389/fonc.2022.1063365

**Published:** 2022-10-20

**Authors:** Lihui Wang, Jian-ye Zhang, Dong-Hua Yang

**Affiliations:** ^1^ Department of Pharmacology, School of Life Science and Biopharmaceutics, Shenyang Pharmaceutical University, Shenyang, China; ^2^ Guangzhou Municipal and Guangdong Provincial Key Laboratory of Molecular Target & Clinical Pharmacology, the National Medical Products Administration (NMPA) and State Key Laboratory of Respiratory Disease, School of Pharmaceutical Sciences and the Fifth Affiliated Hospital, Guangzhou Medical University, Guangzhou, China; ^3^ St. John’s University College of Pharmacy and Health Sciences, Jamaica, NY, United States

**Keywords:** RNA, RNA modification, cancer, diagnosis, treatment

Ribonucleic acid (RNA) is a polymeric molecule that can be found in the vast majority of living organisms and viruses (1). Recent findings have highlighted that RNA modifications are characterized in mRNA and various non-coding RNAs (ncRNAs) (2),whereas they were previously mostly identified in transfer RNA and ribosomal RNA (3). Moreover, the findings of numerous studies have revealed the critical role of non-coding RNAs, such as long ncRNA (lncRNA) (Chen et al.), circular RNA (circRNA) (Ou et al.) small interfering RNAs (siRNAs),and microRNAs (miRNA) (Jia et al.). Dysregulation of RNA modifications caused by an aberrant expression of or mutations is systematically altered and unregulated, which in turn contributes to the initiation, development, or metastasis of various types of cancers (4). Recent advances in the understanding of the functional role of coding and non-coding RNA modifications in tumorigenesis demonstrate that altered RNA biogenesis contributes to controlling the mechanisms of gene expression regulation in cancer (5). More than 170 modification types and millions of modification sites have been identified in all classes of RNA ribonucleotides (6, 7). The recently seminal studies described that some RNA modification focus on N6-methyladenosine (m6A) (Zhang et al.), RNA acetylation (Zhang et al.), pseudouridine (Ψ), 5-methylcytosine (m5C) and N1-methyladenosine (m1A) (6) and their physio pathological functions in cancer (Zhu et al.). The precise method by which RNA alteration influences cancer occurrence and prognosis is, however, still unclear and in the early phases of research. An insight into the technical details of current RNA modification mapping approaches (8), animal models (Guo et al.), and targeting dysregulated RNA modification regulators may help us to discern whether and how these modifications influence cancer. Specific RNA molecules also have the potential to serve as therapeutic agents to develop more effective and personalized treatment strategies for human cancer diseases.

This Research Topic was aimed at updating the basic, translational, and clinical research on the biological and functional roles of RNA modification in various aspects of cancers (**Figure 1**). In general, this topic gathers different contributions highlighting novel findings that will help us shed light on the mechanisms of RNA modification in combating cancer-related diseases.

**Figure 1:**
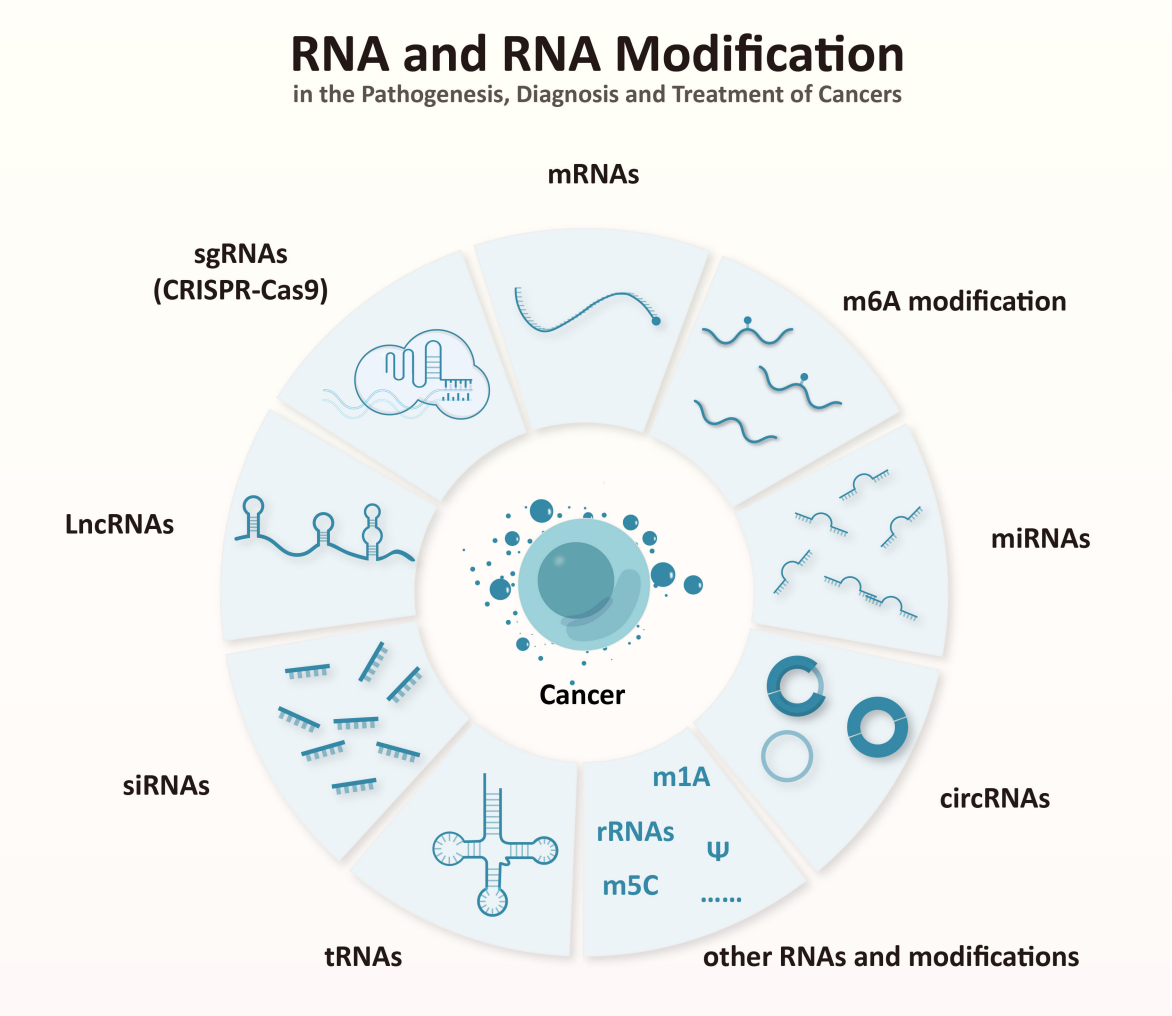
RNA and RNA modifications related to cancer in the topic.

Following the in-depth study of tumors, ncRNA has been emerging as a key regulators of gene expression in carcinogenesis and has gained increasingly more attention and studies. Dysregulated ncRNAs are closely related to promoting and/or suppressing cancer, affecting cancer progression and patient prognosis (Song et al., Farah Ramadan et al.). For example, MicroRNAs (miRNAs) are typically 20–23 nucleotides in length, which was first identified in Caenorhabditis elegans (9). miRNAs participate in many different specific pathways or physiological processes. Ren et al. concluded by reviewing the characteristics of the dual role of miR-149-5p in different cancers that it may be a useful tool for cancer diagnosis or treatment, especially in reproductive system cancers and digestive system cancers.Therefore, it is crucial to clarify the role of miRNAs in cancer pathogenesis and progression. Jia et al. proposed that miR-484 expression in serum and tissues is anticipated as a biomarker and therapeutic target in malignant tumors. Besides the pivotal roles of oncogenes or suppressors in human cancer, miRNAs are also involved in tumor resistance or recurrence through regulatory mechanisms of miRNAs by targeting different genes at multiple stages of autophagy (Lei et al.). Meanwhile, by suppressing oncogene expression, miRNAs (e.g., miR-29b-3p) may be a potential sensitizer of radiation killing in cancer stem cells (CSCs)-like cells (Pan et al.). Circular RNAs (CircRNAs) are another type of non-coding RNA that is typically formed by a type of splicing known as “back-splicing” from a single pre-mRNA (4). Moreover, circular RNA circLMO1 (Hsa_circ_0021087), which has been recognized to have a tumor suppressor effect in many studies, can suppress cervical cancer by triggering miR-4291/acyl-CoA synthetase long chain family member 4 (ACSL4)-mediated ferroptosis (Ou et al.).

Long non-coding RNAs (lncRNAs) are ncRNAs over 200 nucleotides in length (4, 10) and do not encode a protein. Additionally, lncRNA have been shown to engage in a wide range of tumors *via* different mechanisms (Ghafouri-Fard). For example, Ye et al. confirmed the importance of gefitinib metabolism-related lncRNA evaluation in non-small cell lung cancer (NSCLC) patients and identified 13 gefitinib metabolic lncRNA-related prognostic models for NSCLC patients to accurately predict the overall survival of patients.In addition, another study has shown that the different types of lung cancer show distinct features of mRNAs and lncRNAs in the plasma of NSCLC patients. Therefore, these significant plasma biomarkers could have potential value in NSCLC diagnosis (Li et al.). Liu et al. systematically summarized the mechanism of many kinds of lncRNAs, including lncRNA X inactive specific transcript (XIST) as well as their potential therapeutic value in oral cancer. Similarly, LncRNA MDFIC-7 can regulate the miR-525-5p/ARF6 axis, which would promote chordoma progression (Zhang et al.). Several studies in this Research Topic have also investigated the important role of LncRNAs in cancer drug resistance.The upregulation of lncRNA FEZ family zinc finger 1 antisense RNA 1 (FEZF1-AS1) can be associated with poor prognosis of gastric cancer (11). Gui et al. further found that the lncRNA FEZ family zinc finger 1 antisense RNA1 (FEZF1-AS1) also participates in 5-fluorouracil (5-FU) chemo-resistance of GC (Gastric cancer) cells *in vivo* by modulating autophagy.

Dysregulated m6A has been reported to play a significant role in cancer, which can affect the prognosis of patients and the progression of the cancer (Liu et al., Tan et al.). Much evidence has proved that m6A modification participates in tumor malignant progression (Zhou et al.), oncogenic protein expression, multidrug resistance (Chen et al.), stem cell fate (Jiand Zhang) and immune-microenvironment. As a result, it is critical to understand the role of m6A in cancer pathogenesis and progression. Several studies on our topic revealed this effect in various cancers. Zhan et al. reviewed targeting m6A modification as a promising immunotherapeutic approach for turning cold tumors into hot ones that can sensitize cancers to immunotherapy. Similarly, Yu et al. constructed a novel m6A-associated lncRNAs signature that was a strong predictor of immuno-therapeutic responses for the prognosis of pancreatic cancer. Lu et al. found the prognostic value of METTL16 (m6A writer) in pancreatic ductal adenocarcinoma. They suggest that it can modulate the microenvironment, which may become a new target for immunotherapy. Li et al. presented the transcriptome-wide m6A modifications of endometrioid ovarian cancer. MeRIP-seq enabled the discovery of differentially expressed genes with diverse methylated m6A alterations, which further demonstrated the critical oncogenic role of m6A in endometrioid ovarian cancer.

This Research t\Topic also sheds light on additional RNA modification-related cancer risk factors. Xu et al. focused on the 5 RBP-related mRNAs to develop and validate the prognostic model for Hepatitis B virus (HBV) related hepatocellular carcinoma. As discussed in Zhong et al., based on data mining and biological experiments, high mobility group box 3 (HMGB3) expression is abnormally high in neuroblastoma patients with a poor prognosis, which can promote cancer through regulating TPX2. Inhibition of HMGB3 can suppress numerous associated oncogenes, and HMGB3 may represent an excellent novel therapeutic strategy for neuroblastoma (NB).

In summary, RNA-related cancer research is a rapidly emerging field of biotherapeutics, and several RNA therapeutics have been developed for human cancer. In this topic, the authors’ research and summaries show us a promising future in which RNA-based therapeutic approaches have been created as a prospective strategy for a possible cure for human cancer.

## Author contributions

All authors listed have made a substantial, direct, and intellectual contribution to the work and approved it for publication.

## Funding

This work was supported by the National Natural Science Foundation of China (82073320, 81903112), the Central Guidance on Local Science and Technology Development Fund of Liaoning Province (2022JH6/100100038), the “Xingliao Talents” Program of Liaoning Province (XLYC1902008) and Natural Science Foundation of Shenyang (22-315-6-11).

## Conflict of interest

The authors declare that the research was conducted in the absence of any commercial or financial relationships that could be construed as a potential conflict of interest.

## Publisher’s note

All claims expressed in this article are solely those of the authors and do not necessarily represent those of their affiliated organizations, or those of the publisher, the editors and the reviewers. Any product that may be evaluated in this article, or claim that may be made by its manufacturer, is not guaranteed or endorsed by the publisher.
